# Association of miRNA-17-92 Cluster with Muscle Invasion in Bladder Cancer

**DOI:** 10.3390/ijms26157546

**Published:** 2025-08-05

**Authors:** Mihai Ioan Pavalean, Maria Dobre, Iulia Andreea Pelisenco, Victor Lucian Madan, Elena Milanesi, Mihail Eugen Hinescu

**Affiliations:** 1 Faculty of Medicine, Carol Davila University of Medicine and Pharmacy, 050474 Bucharest, Romania; pavaleanmihai21@gmail.com (M.I.P.); victmad@gmail.com (V.L.M.);; 2 Emergency University Central Military Hospital, 010825 Bucharest, Romania; 3 Victor Babeș National Institute of Pathology, 050096 Bucharest, Romaniapelisenco.iulia@gmail.com (I.A.P.)

**Keywords:** bladder cancer, miRNAs, miRNA-17-92 cluster, muscle invasion, risk of progression

## Abstract

Bladder cancer (BC) is the most frequent cancer of the urinary system and one of the most common malignancies in the world. In the last decade, many studies have been conducted to better understand the pathophysiological mechanisms of BC to find innovative markers for disease monitoring and treatment. In this study, we aim to identify miRNAs whose expression is associated with specific tumoral characteristics and risks of disease progression. Forty-one BC patients were enrolled in this study. The expression of 84 miRNAs was evaluated by qRT-PCR analysis on tumoral and peritumoral tissues. The results highlighted the association of the miRNA-17-92 cluster with BC, with miR-17-5p, miR-18a-5p, miR-19a-3p, and miR-20a-5p (members of this cluster) being upregulated in the tumoral tissue and correlated with muscle invasion and tumor grading. Taken together, our study identified a panel of 26 dysregulated miRNAs in BC, some of which may be associated with aggressiveness and the risk of progression of this malignancy.

## 1. Introduction

Bladder cancer (BC) is one of the most widespread cancers worldwide and the most common malignancy of the urinary tract, with more than 90% being urothelial carcinomas [1]. According to GLOBOCAN 2022, BC was the 9th most commonly diagnosed cancer globally. Referring to data specific to gender, new cases of BC in males are estimated to be 5.4% of all new cancer cases in men worldwide, ranking it as the 6th most prevalent cancer in men [2,3]. It constitutes a spectrum of diseases, from recurrent noninvasive tumors managed chronically to aggressive or advanced-stage disease that requires aggressive and multimodal treatment [4]. In the last decade, numerous studies have focused on investigating the pathophysiological mechanisms of BC and finding new strategies for innovative markers for disease monitoring and treatment.

The incidence rate of BC is considerably higher in men than in women, and the epidemiology of this tumor is influenced by the exposure to several carcinogens, tobacco smoking, and the geographical region [2].

About 75% of patients with BC present with non-muscle-invasive bladder cancer (NMIBC) with high rates of recurrence [5]. NMIBC presenting tumoral stages (T) Ta and T1 can benefit from transurethral resection of bladder tumor (TURBT), in some cases supplemented with intravesical instillations to prevent the recurrence and progression of the disease. Muscle-invasive bladder cancer (MIBC) with advanced stages (T  ≥  T2) implies a comprehensive treatment based on radical cystectomy [1]. The 5-year overall survival for T1 is 75%, that for T2 is 50%, and that for T3 is 20% [6].

The BC recurrence and progression risk are very important features when choosing the treatment approach. For this reason, the use of prognostic models, which allow the stratification of patients into risk groups, are vital [7,8,9]. There are many risk stratification and scoring models which use tumor number, size, stage, grade, and status (primary or recurrent, including time to recurrence) to predict recurrence and progression of BC [5]. One such model is the European Association of Urology Non-Muscle-Invasive Bladder Cancer (EAU NMIBC) risk calculator, which aims to offer useful suggestions for the clinical care of NMIBC with an emphasis on clinical presentation and recommendations [10]. Patients with NMIBC have an overall high disease prevalence, expected long-term survival, and a lower risk of disease-specific mortality compared to patients with MIBC [11].

The most common symptom of BC is microscopic or gross hematuria. Gross hematuria is painless, present through urination in 80% of BC patients, and was associated with a higher stage of disease at diagnosis than microscopic hematuria [12,13]. The diagnosis is made during evaluation for microscopic hematuria or, less commonly, pyuria in most asymptomatic patients.

MicroRNAs (miRNAs) can regulate gene expression and control several biological pathways [14]. A single miRNA may target hundreds of mRNAs, while one mRNA can be regulated by multiple miRNAs. Altered miRNA levels may cause the disruption of their target gene activities, affecting multiple molecular pathways leading to tumor initiation and progression [15]. Given their oncogenic or tumor suppressor functions, miRNAs can be valuable candidates for diagnosis and prognosis [16]. Finding the differentially expressed miRNAs in various cancers may help understand the miRNA-mediated pathways and, subsequently, give direction in the treatment [17]. Furthermore, from a practical point of view, due to the short sequence of miRNAs, they are more stable than mRNAs, remaining unaltered during surgical resection procedures [16,18]. Growing evidence supports the important role of miRNAs as prognostic predictors in BC progression, with proliferation and metastasis being key hallmarks that have been extensively investigated in various studies [19].

In this study, we aimed to identify the expression level of a panel of miRNAs in both peritumoral and tumoral BC tissues and to identify possible associations with tumoral characteristics, mainly muscle invasion, grading, and risk of progression.

## 2. Results

In the case–control analysis comparing 41 tumoral vs. 28 peritumoral samples, we found a panel of 26 miRNAs differentially expressed between the two groups. The paired analysis of 24 samples confirmed the differential expression of 25 miRNAs out of 26 (Table 1).

Out of these 26 miRNAs to be found significant dysregulated in our study (Table 1), 24 miRNAs were confirmed in an independent dataset (GSE40355) conducted in bladder urothelial carcinoma tissue vs. normal bladder tissue samples [20]. Furthermore, 22 miRNAs presented the same trend of expression, while 2 miRNAs (let-7c and let-7i) exhibited an opposite trend (data reported in Supplementary Table S2).

**Figure 1:**
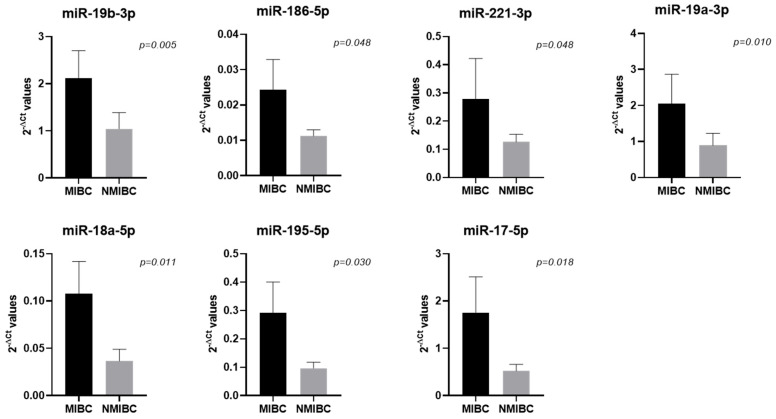
miRNAs found to be differentially expressed between samples with muscle-invasive bladder cancer (MIBC; N = 7) and those with non-muscle-invasive bladder cancer (NMIBC; N = 25). Bar graphs represent the mean of the 2^−ΔCt^ values, and error bars represent the standard error. Statistical significance between the two BC groups was calculated using the Mann–Whitney test.

When investigating the miRNAs’ expression in relation to the tumor characteristics, we found that seven miRNAs were upregulated in tumors showing muscle-invasiveness compared to those without (Table 2, Figure 1).

Moreover, miR-17-5p was found to be more expressed in the patients with recurrent BC compared to those with primary tumor (FR = 2.44, *p* = 0.046).

**Figure 2:**
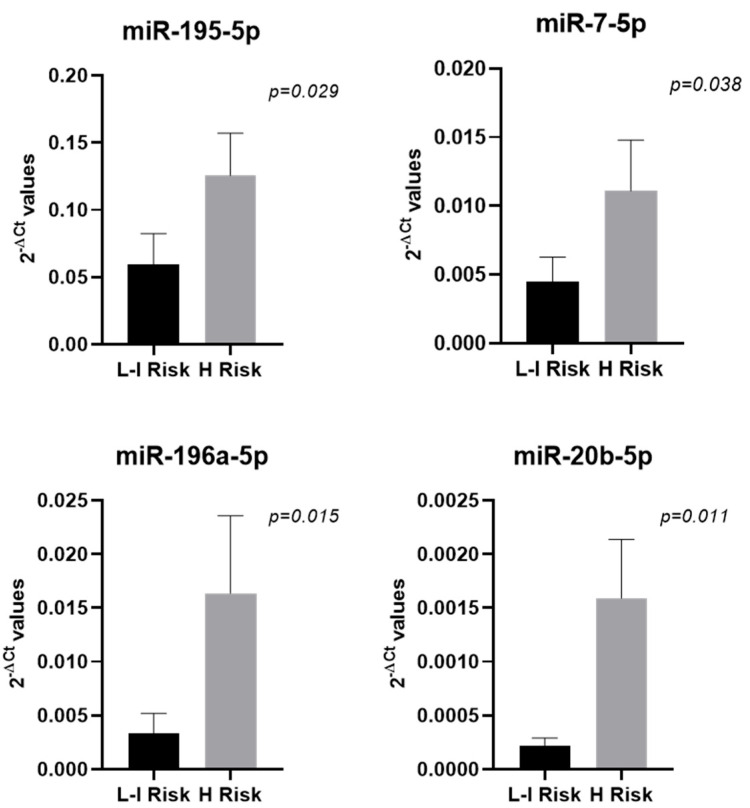
miRNAs found differentially expressed in non-muscle-invasive BC samples comparing tumors with low and intermediate risk (L-I Risk; N = 11) vs. high risk (H Risk; N = 14). Bar graphs represent the mean of the 2^−ΔCt^ values, and error bars represent the standard error. Statistical significance between the two BC groups was calculated using the Mann–Whitney test.

Given that at least 70% of the patients with BC present the non-muscle-invasive subtype, an accurate risk-stratification algorithm that could help establishing treatment strategy and surveillance schedules was recommended by the European Association of Urology (EAU) 1. The analysis of the short- and long-term risks of disease progression, performed on the 25 patients presenting non-muscle-invasive phenotype, revealed that four patients (16.00%) had low risk, seven patients (28.00%) intermediate risk, and fourteen patients (56%) high risk. Due to the low frequency of patients presenting low and intermediate risk in our cohort, we stratified the samples in two groups, low–intermediate risk (N = 11) and high risk (N = 14), and the miRNA profile between these two groups was compared. We found four miRNAs to be significantly upregulated (FR > 2, *p* < 0.05) in patients with high risk compared to those with low and intermediate risk (Table 3, Figure 2).

**Figure 3:**
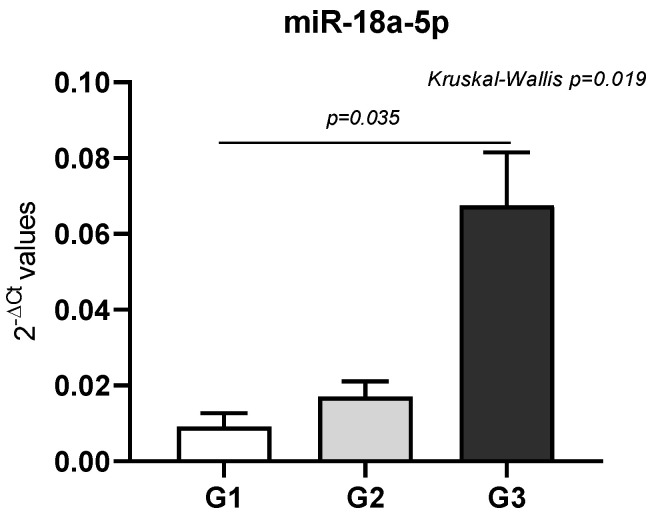
The graph shows miR-18a-5p as differentially expressed among the three BC groups (G1 = grade 1; G2 = grade 2, G3 = grade 3). Bar graphs represent the mean of the 2^−ΔCt^ values, and error bars represent the standard error. The *p* value has been calculated using Kruskal–Wallis test followed by pairwise comparisons using Dunn’s test.

Additionally, given that miRNAs’ expression level may influence the tumor grade, we found a significant association between miR-18a-5p and the grading tumor (*p* = 0.019) as shown in Figure 3.

## 3. Discussion

Bladder cancer (BC) remains the most prevalent malignancy affecting the urinary system and one of the most widespread cancers worldwide [21]. In the last decade, numerous studies were focused on investigating the involvement of miRNAs in BC development and their potential role as candidates for diagnosis and prognosis [22].

Understanding cancer pathways from a molecular perspective has gained attention in recent years, and miRNA expression has emerged as a promising research strategy.

In this study, we evaluated, through qRT-PCR analysis, the expression level of 84 miRNAs in tumoral tissues from 41 patients diagnosed with BC vs. peritumoral tissue from 28 patients. Peritumoral tissue, confirmed by a pathologist to lack cancer cells, was considered as a control group, given that normal bladder tissue was not available for the case–control analyses. When performing case–control study, the statistical analysis revealed that 26 miRNAs were differentially expressed. After performing paired analysis on 24 paired samples, out of the 26 miRNAs previously identified, 25 miRNAs showed a significant dysregulation. Our result is supported by a previous microarray analysis (GSE40355) where 24 miRNAs (out of the 26 we identified) were found to be significantly dysregulated in bladder urothelial carcinoma compared to normal bladder tissue samples (data reported in Supplementary Table S2) [20]. Moreover, 15 miRNAs were downregulated in tumoral tissue in both case–control and paired analysis, most of them being described as tumor-suppressor miRNAs in BC [23]. Several studies reported that miR-143 and miR-1 can suppress BC cell proliferation, invasion, and migration [16,24,25]; furthermore, miR-214, miR-145, and miR-126 can be used as potential biomarkers for early diagnosis, prognosis, and recurrence of BC [26,27]. MiR-143 and miR-145 form a bicistronic cluster and have tumor suppressor functions and could be helpful in BC outcome prediction, given their association with BC aggressiveness [24].

When conducting further analysis to find possible association between the expression level of miRNAs and the tumor features, we found a panel of miRNAs that seem to be associated with muscle invasion, risk of disease progression, and tumor grading.

Notably, four of the dysregulated miRNAs highlighted in the present study (miR-17-5p, miR-18a-5p, miR-19a-3p, and miR-19b-3p) that were found to be upregulated in the case–control comparison belong to the miR-17-92 cluster. The miR-17-92 cluster is located on Chr 13q31.3 and, following transcription, results a primary transcript which encodes six distinct mature miRNAs: miR-17, miR-18a, miR-19a, miR-19b, miR-20a, and miR-92a [28]. This chromosomal location is amplified in hematological malignancies, and overexpression of the miR-17-92 cluster has been found in a wide range of tumors—BC, lung cancer, pancreatic cancer, prostate cancer, lung cancer—being called “oncomiR-1”, since several reports have demonstrated its oncogenic function [29,30,31,32].

miR-17-5p is involved in a variety of biological processes and it has been found to promote proliferation and invasion in a wide range of cancers [33,34,35]. In our study, miR-17-5p was upregulated in tumors with muscle invasion compared to those without. In line with our findings, Hejia Yuan et al. reported high levels of miR-17-5p in muscle-invasive BC compared to non-muscle-invasive BC [36]. In addition, in our study, miR-17-5p was found to be more expressed in the patients with recurrent BC compared to those with primary tumor. In line with this result, high levels of miR-17-5p have been found to be associated with recurrence in patients with prostate cancer and in those with head and neck squamous cell carcinoma [37,38].

Another miRNA that was found to be associated with muscle invasion and BC grading is miR-18a-5p. This miRNA is well-known to play a key role in regulating cancer metastasis and other important processes such as cell proliferation and migration [39]. According to our results, mir-18a-5p expressed higher levels in patients with muscle invasion BC compared to those without. The same trend of increased levels of miR-18a-5p has been reported by others when comparing BC tissues to peritumoral tissues [39,40,41]. When we stratified the patients based on the tumor grading, higher levels at G3 than G1 and G2 were found. To our knowledge, up to now, no previous studies have reported an association between the levels of miR-18a-5p and the tumor grade.

The dysregulation of miR-19a-3p in BC and in other tumors such as gastric, prostate, and breast cancer has been reported before, along with its effect on BC progression [42,43,44,45]. In the present study, miR-19a-3p displayed higher levels when comparing sample of BC with muscle invasion and without. miR-19a-3p together with miR-19b-3p were reported to be upregulated in BC, both in formalin-fixed paraffin-embedded tissue and fresh-frozen tumoral tissues, using deep sequencing and microarray methods, respectively [46,47]. This miRNA also plays a pivotal role in proliferation and migration of lung cancer [48], and its levels positively correlate with tumor size, lymph node metastasis, and clinical stage in esophageal squamous-cell carcinoma [49]. When we focused the analysis which compared BC with muscle invasion and without, we found that miR-19b-3p was upregulated in the first group.

Beside the miRNAs belonging to the miRNA-17-92 cluster found to be associated with muscle invasion and grading, in this study, another panel of four miRNAs was found to be associated with risk of BC progression in NMIBC patients. This panel comprise miR-7-5p, miR-20b-5p, miR-195-5p, and miR-196-5p. According to the last available review on the role of miRNAs in predicting the risk of progression in NMIBC, none of the miRNAs identified in our study has been previously reported to be associated with this feature [50]. This could be due to the differences in sociodemographic data, clinical features, and tumor characteristics across the investigated cohorts. Extensive studies conducted in larger cohort of NMIBC patients is required, which will allow the usual stratification into three groups: low risk, intermediate risk, and high risk.

Some limitations of the present study must be noted: (i) the number of individuals involved in the study is small, leading to the need of validate the results in large cohort to obtain a better statistical power; (ii) normal bladder tissue was not available for the case–control analyses and the findings refer to the comparison with peritumoral tissue, which does not represent an ideal control; (iii) stratification of the NMIBC patients in two groups, low–intermediate risk and high risk, due to the reduced number of patients with low and intermediate risk in our cohort; (iv) while qRT-PCR is a common method for analyzing miRNAs levels, the results could be also confirmed with more sensitive techniques, such as digital PCR.

## 4. Materials and Methods

### 4.1. Sample Collection

In this study, 41 patients with a diagnosis of bladder cancer (BC) were enrolled at the Department of Urology, Emergency University Central Military Hospital, Bucharest, from December 2022 to December 2023; ethical approval was obtained (Approval nr. 556/20 December 2022), and written informed consent was obtained from all individuals in agreement with the Helsinki Declaration of 1975 as revised in 2013. None of the patients underwent any type of treatment, such as intravesical therapy or neoadjuvant chemotherapy, before sample collection. For 36 patients, the tumoral tissue was collected after TURBT, while 5 individuals underwent cystectomy. Out of the 41 patients enrolled in this study, 29 patients were diagnosed with BC for the first time, while 12 presented a recurrent disease.

A total of 25 patients were included in the NMIBC group: 15 patients with Ta stage, 3 patients with T1 stage with muscularis propria in the resected specimen, and 7 patients with T1 stage without residual disease and with muscularis propria in the re-resected specimen at the evaluation performed 30–42 days after the first resection. Nine patients were diagnosed with T1 stage; however, since the muscularis propria was absent in the resected specimen and they did not perform the re-evaluation in the mentioned hospital, these were not included in the NMIBC group. Seven patients were diagnosed with MIBC. For patients included in the NMIBC group, the short- and long-term risks of disease progression been evaluated using the EAU NMIBC risk calculator (https://www.nmibc.net/ (accessed on 16 Decembre 2024)).

In the absence of normal bladder tissue for the case–control analyses, the control group was represented by 28 peritumoral tissues obtained after TURBT. The lack of cancer cells in the peritumoral samples was confirmed by a pathologist.

All 41 patients included in this study were considered in the case–control analysis, while 24 patients who presented both tumoral (T) and peritumoral tissue (PT) were considered a subgroup for a paired analysis. The sociodemographic, clinical, and tumor records of the cohort are reported in Table 4.

### 4.2. miRNAs Expression Analysis and Omnibus Dataset Analysis

After resection, the tumoral and peritumoral tissue specimens were preserved in RNAprotect Tissue Reagent (Qiagen, Hilden, Germany) for 48–72h to avoid RNA degradation and, after being removed, the samples were stored at −80 °C until total RNA isolation. The miRNeasy Mini Kit (Qiagen, Hilden, Germany) was used according to the manufacturer’s instructions to isolate total RNA, including miRNAs. Using the spectrophotometric method, the NanoDrop 2000 (Thermo Fisher Scientific, Inc., Waltham, MA, USA) was used to assess RNA quality and quantity. All samples had both 260/280 nm and 260/230 nm ratio >1.8. A miRCURY LNA RT Kit (Qiagen, Hilden, Germany) was used to reverse transcribe 10 ng of total RNA by the polyadenylation-based method according to the manufacturer’s protocol. The expression of 84 miRNAs included in the Human Cancer Focus YAHS-102, miRCURY LNA miRNA Focus PCR Panel (Qiagen, Hilden, Germany), a validated array for different types of human cancer, was evaluated using the miRCURY LNA SYBR Green PCR Kit (Qiagen, Hilden, Germany) by qRT-PCR analysis on an ABI-7500 fast instrument (Thermo Fisher Scientific, Inc., Waltham, MA, USA). Three miRNAs (miR-149-3p, miR-202-3p, and miR-206-3p) were excluded from the analysis of all samples since their Ct values were above 35 and they were therefore considered undetected.

Among the three putative reference RNAs (SNORD38B, SNORD49A, and U6 snRNA) suggested by the provider of the kit, using the RefFinder algorithm (https://www.ciidirsinaloa.com.mx/RefFinder-master/ (accessed on 12 Decembre 2024).), we identified SNORD38B and SNORD49A as the most stable, and therefore their geometric mean values were used for Ct normalization to obtain ∆Ct values. The microRNA name, the target sequences, and the corresponding LNA™ microRNA PCR primer set (Prod No) are reported in Supplementary Table S1. The miRNA expression statistical analysis was performed on the 2^−∆Ct^ values. Fold Change (FC) was calculated as 2^−ΔΔCT^ values. When the FC value was above 1, fold regulation (FR) was equal to FC; when the FC value was less than 1, FR was expressed as the negative inverse of FC. In the results section, data are shown as both fold regulation (FR) and as 2^−∆Ct^ values.

The miRNAs found to be significantly dysregulated in our study, when comparing 41 T vs. 28 PT and 24 T vs. 24 PT, were searched in an independent dataset. In the NCBI GEO database, we identified one dataset (GSE40355) that analyzed miRNA expression in 16 urothelial carcinoma and 8 normal bladder tissue samples [20]. This dataset was downloaded and reanalyzed using GEO2R for differential expression, considering the comparison urothelial carcinoma vs. normal bladder tissue.

### 4.3. Statistical Analysis

Since the levels of miRNA were not normally distributed (Shapiro–Wilk test, *p*  <  0.05), non-parametric tests were applied. The Mann–Whitney test was used in the case–control study and in MIBC vs. NMIBC comparison, while the Wilcoxon signed-rank test was applied to assess the differences between paired tumoral and peritumoral tissues. When more than two groups were compared, the Kruskal–Wallis test, followed by pairwise comparisons using Dunn’s test with Bonferroni correction, was performed. The Statistical Package for the Social Sciences (SPSS version 20.0) and the GraphPad Prism 8.4.3. were used to perform statistical analysis and generate the graphs, respectively. The difference in miRNAs levels between the different tested groups was considered significant when *p*  <  0.05 and −2  ≥  FR  ≥  2.

## 5. Conclusions

Taken together, our study demonstrates the differential expression of a panel of 26 miRNAs in BC, with a particular association of four miRNAs belonging to the miRNA-17-92 cluster with muscle invasion and tumor grading. Further analysis conducted in large cohort is needed to validate the potential of these miRNAs as biomarkers in BC.

## Figures and Tables

**Table 1:** miRNAs differentially expressed in the case–control (Mann–Whitney test) and paired (Wilcoxon test) analyses. The miRNAs are displayed based on their FR values, from lowest to highest.

miRNA	Case–Control (41 T vs. 28 PT)				Paired (24 T vs. 24 PT)			
	2^−∆Ct^ (Mean ± SD) T	2^−∆Ct^ (Mean ± SD) PT	FR	*p*-Value	2^−∆Ct^ (Mean ± SD) T	2^−∆Ct^ (Mean ± SD) PT	FR	*p*-Value
**miR-133a-3p**	0.015 ± 0.04	0.197 ± 0.22	**−13.49**	<0.001	0.020 ± 0.05	0.189 ± 0.21	**−9.53**	<0.001
**miR-1**	0.012 ± 0.03	0.113 ± 0.14	**−9.20**	<0.001	0.017 ± 0.04	0.114 ± 0.14	**−6.71**	<0.001
**miR-100-5p**	0.091 ± 0.17	0.742 ± 0.45	**−8.12**	<0.001	0.109 ± 0.20	0.735 ± 0.48	**−6.72**	<0.001
**miR-99a-5p**	0.088 ± 0.17	0.702 ± 0.48	**−7.95**	<0.001	0.103 ± 0.28	0.708 ± 0.51	**−6.86**	<0.001
**miR-125b-5p**	0.743 ± 1.68	5.307 ± 3.01	**−7.15**	<0.001	0.923 ± 2.09	5.408 ± 3.17	**−5.86**	<0.001
**miR-145-5p**	2.458 ± 4.58	16.799 ± 16.04	**−6.84**	<0.001	2.957 ± 5.66	16.722 ± 16.84	**−5.66**	<0.001
**miR-143-3p**	1.030 ± 1.70	5.635 ± 4.82	**−5.47**	<0.001	1.030 ± 1.70	5.635 ± 4.82	**−4.72**	<0.001
**miR-132-3p**	0.019 ± 0.02	0.063 ± 0.05	**−3.37**	<0.001	0.018 ± 0.02	0.062 ± 0.05	**−3.34**	<0.001
**miR-150-5p**	0.353 ± 0.52	1.130 ± 1.53	**−3.20**	<0.001	0.271 ± 0.33	1.209 ± 1.64	**−4.46**	<0.001
**miR-214-3p**	0.129 ± 0.23	0.370 ± 0.18	**−2.86**	<0.001	0.113 ± 0.22	0.369 ± 0.20	**−3.27**	<0.001
**miR-126-3p**	1.158 ± 1.56	2.726 ± 1.61	**−2.35**	<0.001	0.991 ± 0.90	2.713 ± 1.65	−2.74	<0.001
**let-7c-5p**	0.803 ± 0.55	1.889 ± 1.45	**−2.35**	<0.001	0.804 ± 0.56	1.972 ± 1.55	**−2.45**	<0.001
**miR-195-5p**	0.164 ± 0.23	0.355 ± 0.34	**−2.17**	<0.001	0.182 ± 0.28	0.352 ± 0.35	−1.93	0.009
**miR-26a-5p**	1.119 ± 0.90	2.262 ± 1.04	**−2.02**	<0.001	1.267 ± 1.04	2.246 ± 1.02	−1.77	0.002
**let-7i-5p**	0.209 ± 0.22	0.419 ± 0.22	**−2.00**	<0.001	0.209 ± 0.20	0.429 ± 0.23	−2.06	<0.001
**miR-200c-3p**	4.411 ± 3.83	2.149 ± 1.68	**2.05**	0.018	4.130 ± 3.76	1.973 ± 1.46	**2.09**	0.012
**miR-106b-5p**	0.346 ± 0.27	0.162 ± 0.09	**2.13**	0.003	0.328 ± 0.28	0.157 ± 0.08	2.09	0.004
**miR-20a-5p**	0.929 ± 1.05	0.417 ± 0.36	**2.23**	0.011	0.725 ± 0.99	0.397 ± 0.37	ns	ns
**miR-106a-5p**	0.771 ± 0.88	0.332 ± 0.22	**2.33**	0.039	0.711 ± 0.97	0.318 ± 0.21	2.23	0.040
**miR-21-5p**	12.287 ± 11.41	4.902 ± 4.28	**2.51**	0.004	10.189 ± 11.31	4.833 ± 4.40	**2.10**	0.004
**miR-19b-3p**	1.313 ± 1.60	0.478 ± 0.39	**2.75**	0.006	0.962 ± 1.23	0.468 ± 0.41	**2.06**	0.037
**miR-17-5p**	0.810 ± 1.08	0.270 ± 0.29	**3.00**	0.001	0.721 ± 1.26	0.266 ± 0.30	**2.72**	0.005
**miR-19a-3p**	1.142 ± 1.67	0.370 ± 0.40	**3.09**	0.003	0.921 ± 1.48	0.361 ± 0.43	**2.55**	0.012
**miR-18a-5p**	0.052 ± 0.07	0.015 ± 0.02	**3.55**	<0.001	0.041 ± 0.06	0.015 ± 0.02	**2.82**	0.002
**miR-182-5p**	0.172 ± 0.31	0.036 ± 0.06	**4.79**	<0.001	0.196 ± 0.40	0.033 ± 0.05	**5.87**	<0.001
**miR-210-3p**	0.183 ± 0.17	0.034 ± 0.04	**5.38**	<0.001	0.139 ± 0.12	0.034 ± 0.04	**4.14**	<0.001

**Table 2:** miRNAs differentially expressed in MIBC vs. NMIBC samples (Mann–Whitney test). The miRNAs are displayed based on their FR values, from lowest to highest.

miRNA	MIBC (N = 7) 2^−∆Ct^ (Mean ± SD)	NMIBC (N = 25) 2^−∆Ct^ (Mean ± SD)	FR	*p*-Value
**miR-19b-3p**	2.125 ± 1.52	1.043 ± 1.71	**2.04**	0.005
**miR-186-5p**	0.024 ± 0.02	0.011 ± 0.01	**2.16**	0.048
**miR-221-3p**	0.278 ± 0.38	0.126 ± 0.13	**2.21**	0.048
**miR-19a-3p**	2.047 ± 2.16	0.889 ± 1.68	**2.30**	0.010
**miR-18a-5p**	0.108 ± 0.09	0.036 ± 0.06	**2.95**	0.011
**miR-195-5p**	0.291 ± 0.29	0.096 ± 0.10	**3.02**	0.030
**miR-17-5p**	1.756 ± 2.00	0.522 ± 0.68	**3.36**	0.018

**Table 3:** miRNAs differentially expressed in NIMBC samples with low and intermediate risk (L-I Risk) vs. high risk (H Risk) (Mann–Whitney test). The miRNAs are displayed based on their FR values, from lowest to highest.

miRNA	L-I Risk (N = 11) 2^−∆Ct^ (Mean ± SD)	H Risk (N = 14) 2^−∆Ct^ (Mean ± SD)	FR	*p*-Value
**miR-195-5p**	0.059 ± 0.07	0.126 ± 0.12	**2.12**	0.029
**miR-7-5p**	0.004 ± 0.005	0.011 ± 0.013	**2.47**	0.038
**miR-196a-5p**	0.003 ± 0.006	0.163 ± 0.02	**4.94**	0.015
**miR-20b-5p**	0.0002 ± 0.0002	0.0015 ± 0.0020	**7.17**	0.011

**Table 4:** Sociodemographic, clinical, and tumor features of the enrolled cohort.

	Total (N = 41)		NMIBC (N = 25)		MIBC (N = 7)	
**Sociodemographic Data and Comorbidities**						
**Sex (%F; %M)**	22%; 78%		24%; 76%		28.6%; 71.4%	
	Mean ± SD	min–max	Mean ± SD	min-max	Mean ± SD	min-max
**Age**	69.61 ± 10.04	39–85	68.04 ± 11.42	39–85	70.29 ± 4.71	64–78
**BMI**	29.21 ± 6.65	20.44–55.10	30.54 ± 7.35	20.44–55.10	26.13 ± 3.85	20.76–32.79
	available for 38 patients					
	**% YES**	**% NO**	**% YES**	**% NO**	**% YES**	**% NO**
**Smoker**	48.60%	51.40%	48%	52%	57.10%	42.90%
	available for 37 patients					
**Obesity**	31.60%	68.40%	40%	60%	14.30%	85.70%
	available for 38 patients					
**Hypertension**	42.10%	57.90%	44%	56%	28.60%	71.40%
	available for 38 patients					
**Diabetes**	7.30%	92.70%	4%	96%	0.00%	100%
**Heart failure**	12.50%	87.50%	16%	84%	14.30%	85.70%
	available for 40 patients					
**Coronary heart disease**	2.50%	97.50%	0.00%	100%	0.00%	100%
	available for 40 patients					
**Dyslipidemia**	25%	75%	36%	64%	0.00%	100%
	available for 40 patients					
**Biochemical parameters**						
	**mean ± SD**	**min–max**	**mean ± SD**	**min–max**	**mean ± SD**	**min–max**
**Hemoglobin (g/dL)**	12.71 ± 2.87	3.8–16.1	13.36 ± 2.20	7.9–16.1	10.61 ± 3.81	3.8–15.6
**Glycemia (mg/dL)**	121.48 ± 31.62	74–208	113.63 ± 31.56	83.0–208.0	123.14 ± 26.07	74.0–146.0
	available for 40 patients		available for 24 patients			
**White blood cells**	7.99 ± 2.54	4.05–16.78	8.07 ± 2.68	4.05–16.78	7.52 ± 2.23	4.14–9.96
**(N × 10^3^/µL)**						
**Platelets**	257.29 ± 73.99	82–432	273.92 ± 61.32	193.0–432.0	246.57 ± 101.94	82.0–394.0
**(N × 10^3^/µL)**						
**Lymphocytes**	1.81 ± 0.68	0.84–3.61	1.94 ± 0.76	0.84–3.61	1.66 ± 0.57	0.92–2.41
**(N × 10^3^/µL)**						
**Neutrophile**	5.14 ± 2.19	1.34–12.51	4.97 ± 2.27	1.34–12.51	5.15 ± 2.06	2.32–745.0
**(N × 10^3^/µL)**						
**Tumor Characteristics**						
**Recurrence**	Primary 73.2%		Primary 80%		Primary 28.6%	
	Recurrent 26.8%		Recurrent 20%		Recurrent 71.4%	
**Grade**	G1 12.2%		G1 20%			
	G2 17.1%		G2 20%		G2 14.3%	
	G3 70.7%		G3 60%		G3 85.7%	
**TNM classification**	Ta 36.6%		Ta 60%			
	T1 46.3%		T1 40%			
	T2 12.2%				T2 71.4%	
	T4 4.9%				T4 28.6%	

NIMBC: non-muscle-invasive bladder cancer; MIBC: muscle-invasive bladder cancer.

## Data Availability

The data presented in this study are available on reasonable request from the corresponding author.

## Supplementary Materials

The following supporting information can be downloaded at: https://www.mdpi.com/article/10.3390/ijms26157546/s1.

## Abbreviations

The following abbreviations are used in this manuscript:

BC	Bladder cancer
NMIBC	Non-muscle-invasive bladder cancer
TURBT	Transurethral resection of bladder tumor
MIBC	Muscle-invasive bladder cancer
EAU NMIBC	European Association of Urology Non-Muscle-Invasive Bladder Cancer
miRNAs	MicroRNAs

## References

[1] Qin C., Tian Q., Zhou H., Qin Y., Zhou S., Wu Y., Tianjiao E., Duan S., Li Y., Wang X. (2024). Detecting Muscle Invasion of Bladder Cancer: An Application of Diffusion Kurtosis Imaging Ratio and Vesical Imaging-Reporting and Data System. J. Magn. Reson. Imaging.

[2] Bray Bsc F., Laversanne M., Sung H., Ferlay J., Siegel R.L., Soerjomataram I., Jemal A. (2024). Global Cancer Statistics 2022: GLOBOCAN Estimates of Incidence and Mortality Worldwide for 36 Cancers in 185 Countries. CA A Cancer J. Clin..

[3] GLOBOCAN 2022: Bladder Cancer 9th Most Common Worldwide—World Bladder Cancer Patient Coalition. https://worldbladdercancer.org/news_events/globocan-2022-bladder-cancer-is-the-9th-most-commonly-diagnosed-worldwide/.

[4] Lobo N., Hensley P.J., Bree K.K., Nogueras-Gonzalez G.M., Navai N., Dinney C.P., Sylvester R.J., Kamat A.M. (2022). Updated European Association of Urology (EAU) Prognostic Factor Risk Groups Overestimate the Risk of Progression in Patients with Non-Muscle-Invasive Bladder Cancer Treated with Bacillus Calmette-Guérin. Eur. Urol. Oncol..

[5] Grabe-Heyne K., Henne C., Mariappan P., Geiges G., Pöhlmann J., Pollock R.F. (2023). Intermediate and High-Risk Non-Muscle-Invasive Bladder Cancer: An Overview of Epidemiology, Burden, and Unmet Needs. Front. Oncol..

[6] Dziewit A., Sinha A.C. (2024). Bladder Cancer. Essence of Anesthesia Practice.

[7] Gontero P., Sylvester R., Pisano F., Joniau S., Vander Eeckt K., Serretta V., Larré S., Di Stasi S., Van Rhijn B., Witjes A.J. (2015). Prognostic Factors and Risk Groups in T1G3 Non–Muscle-Invasive Bladder Cancer Patients Initially Treated with Bacillus Calmette-Guérin: Results of a Retrospective Multicenter Study of 2451 Patients. Eur. Urol..

[8] Sylvester R.J., Van Der Meijden A.P., Oosterlinck W., Witjes J.A., Bouffioux C., Denis L., Newling D.W.W., Kurth K. (2006). Predicting Recurrence and Progression in Individual Patients with Stage Ta T1 Bladder Cancer Using EORTC Risk Tables: A Combined Analysis of 2596 Patients from Seven EORTC Trials. Eur. Urol..

[9] Fernandez-Gomez J., Madero R., Solsona E., Unda M., Martinez-Piñeiro L., Gonzalez M., Portillo J., Ojea A., Pertusa C., Rodriguez-Molina J. (2009). Predicting Nonmuscle Invasive Bladder Cancer Recurrence and Progression in Patients Treated with Bacillus Calmette-Guerin: The CUETO Scoring Model. J. Urol..

[10] Babjuk M., Burger M., Capoun O., Cohen D., Compérat E.M., Dominguez Escrig J.L., Gontero P., Liedberg F., Masson-Lecomte A., Mostafid A.H. (2022). European Association of Urology Guidelines on Non–Muscle-Invasive Bladder Cancer (Ta, T1, and Carcinoma in Situ). Eur. Urol..

[11] Burger M., Catto J.W.F., Dalbagni G., Grossman H.B., Herr H., Karakiewicz P., Kassouf W., Kiemeney L.A., La Vecchia C., Shariat S. (2013). Epidemiology and Risk Factors of Urothelial Bladder Cancer. Eur. Urol..

[12] Ramirez D., Gupta A., Canter D., Harrow B., Dobbs R.W., Kucherov V., Mueller E., Streeper N., Uhlman M.A., Svatek R.S. (2016). Microscopic Haematuria at Time of Diagnosis Is Associated with Lower Disease Stage in Patients with Newly Diagnosed Bladder Cancer. BJU Int..

[13] Gilligan T.D., Steele G.S., Zietman A.L., Kantoff P.W., Kufe D.W., Pollock R.E., Weichselbaum R.R. (2003). Superficial Bladder Carcinoma. Holland-Frei Cancer Medicine.

[14] Xie Y., Ma X., Chen L., Li H., Gu L., Gao Y., Zhang Y., Li X., Fan Y., Chen J. (2017). MicroRNAs with Prognostic Significance in Bladder Cancer: A Systematic Review and Meta-Analysis. Sci. Rep..

[15] Peng Y., Croce C.M. (2016). The Role of MicroRNAs in Human Cancer. Signal Transduct. Target. Ther..

[16] Braicu C., Buiga R., Cojocneanu R., Buse M., Raduly L., Pop L.A., Chira S., Budisan L., Jurj A., Ciocan C. (2019). Connecting the Dots between Different Networks: MiRNAs Associated with Bladder Cancer Risk and Progression. J. Exp. Clin. Cancer Res..

[17] Hui A., How C., Ito E., Liu F.F. (2011). Micro-RNAs as Diagnostic or Prognostic Markers in Human Epithelial Malignancies. BMC Cancer.

[18] Bartel D.P. (2009). MicroRNAs: Target Recognition and Regulatory Functions. Cell.

[19] Yerukala Sathipati S., Tsai M.J., Shukla S.K., Ho S.Y., Liu Y., Behesthi A. (2022). MicroRNA Signature for Estimating the Survival Time in Patients with Bladder Urothelial Carcinoma. Sci. Rep..

[20] Hecker N., Stephan C., Mollenkopf H.J., Jung K., Preissner R., Meyer H.A. (2013). A New Algorithm for Integrated Analysis of MiRNA-MRNA Interactions Based on Individual Classification Reveals Insights into Bladder Cancer. PLoS ONE.

[21] Dobruch J., Oszczudłowski M. (2021). Bladder Cancer: Current Challenges and Future Directions. Medicina.

[22] Homami A., Ghazi F. (2016). MicroRNAs as Biomarkers Associated with Bladder Cancer. Med. J. Islam. Repub. Iran..

[23] Taheri M., Shirvani-Farsani Z., Ghafouri-Fard S., Omrani M.D. (2020). Expression Profile of MicroRNAs in Bladder Cancer and Their Application as Biomarkers. Biomed. Pharmacother..

[24] Avgeris M., Mavridis K., Tokas T., Stravodimos K., Fragoulis E.G., Scorilas A. (2015). Uncovering the Clinical Utility of MiR-143, MiR-145 and MiR-224 for Predicting the Survival of Bladder Cancer Patients Following Treatment. Carcinogenesis.

[25] Zhang J., Wang L., Mao S., Liu M., Zhang W., Zhang Z., Guo Y., Huang B., Yan Y., Huang Y. (2018). MiR-1-3p Contributes to Cell Proliferation and Invasion by Targeting Glutaminase in Bladder Cancer Cells. Cell. Physiol. Biochem..

[26] Zaravinos A., Radojicic J., Lambrou G.I., Volanis D., Delakas D., Stathopoulos E.N., Spandidos D.A. (2012). Expression of MiRNAs Involved in Angiogenesis, Tumor Cell Proliferation, Tumor Suppressor Inhibition, Epithelial-Mesenchymal Transition and Activation of Metastasis in Bladder Cancer. J. Urol..

[27] Nagata M., Muto S., Horie S. (2016). Molecular Biomarkers in Bladder Cancer: Novel Potential Indicators of Prognosis and Treatment Outcomes. Dis. Markers.

[28] Zhao W., Gupta A., Krawczyk J., Gupta S. (2022). The MiR-17-92 Cluster: Yin and Yang in Human Cancers. Cancer Treat. Res. Commun..

[29] Wang J., Peng X., Li R., Liu K., Zhang C., Chen X., Huang G., Zhao L., Chen Z., Lai Y. (2021). Evaluation of Serum MiR-17-92 Cluster as Noninvasive Biomarkers for Bladder Cancer Diagnosis. Front. Oncol..

[30] Fang L.L., Wang X.H., Sun B.F., Zhang X.D., Zhu X.H., Yu Z.J., Luo H. (2017). Expression, Regulation and Mechanism of Action of the MiR-17-92 Cluster in Tumor Cells (Review). Int. J. Mol. Med..

[31] Mogilyansky E., Rigoutsos I. (2013). The MiR-17/92 Cluster: A Comprehensive Update on Its Genomics, Genetics, Functions and Increasingly Important and Numerous Roles in Health and Disease. Cell Death Differ..

[32] Kurozumi A., Goto Y., Okato A., Ichikawa T., Seki N. (2016). Aberrantly Expressed MicroRNAs in Bladder Cancer and Renal Cell Carcinoma. J. Hum. Genet..

[33] Song J., Liu Y., Wang T., Li B., Zhang S. (2020). MiR-17-5p Promotes Cellular Proliferation and Invasiveness by Targeting RUNX3 in Gastric Cancer. Biomed. Pharmacother..

[34] Selven H., Andersen S., Pedersen M.I., Lombardi A.P.G., Busund L.T.R., Kilvær T.K. (2022). High Expression of MiR-17-5p and MiR-20a-5p Predicts Favorable Disease-Specific Survival in Stage I-III Colon Cancer. Sci. Rep..

[35] Chen C., Lu Z., Yang J., Hao W., Qin Y., Wang H., Xie C., Xie R. (2016). MiR-17-5p Promotes Cancer Cell Proliferation and Tumorigenesis in Nasopharyngeal Carcinoma by Targeting P21. Cancer Med..

[36] Yuan H., Wang T., Peng P., Xu Z., Feng F., Cui Y., Ma J., Wu J. (2024). Urinary Exosomal MiR-17-5p Accelerates Bladder Cancer Invasion by Repressing Its Target Gene ARID4B and Regulating the Immune Microenvironment. Clin. Genitourin. Cancer.

[37] Stoen M.J., Andersen S., Rakaee M., Pedersen M.I., Ingebriktsen L.M., Bremnes R.M., Donnem T., Lombardi A.P.G., Kilvaer T.K., Busund L.T. (2021). High Expression of MiR-17-5p in Tumor Epithelium Is a Predictor for Poor Prognosis for Prostate Cancer Patients. Sci. Rep..

[38] Huang Q., Shen Y.J., Hsueh C.Y., Guo Y., Zhang Y.F., Li J.Y., Zhou L. (2021). MiR-17-5p Drives G2/M-Phase Accumulation by Directly Targeting CCNG2 and Is Related to Recurrence of Head and Neck Squamous Cell Carcinoma. BMC Cancer.

[39] Kolenda T., Guglas K., Kopczyńska M., Sobocińska J., Teresiak A., Bliźniak R., Lamperska K. (2020). Good or Not Good: Role of MiR-18a in Cancer Biology. Rep. Pract. Oncol. Radiother..

[40] Zhang Z., Liu T., Cheng C., Wang J., Wang C., Huang H., Li Y. (2022). LncRNA GAS5 Regulates the Wnt/β-Catenin Pathway through the MiR-18a-5p/AXIN2/GSK3β Axis to Inhibit the Proliferation and Migration of Bladder Cancer Cells. Carcinogenesis.

[41] Li J.H., Liu S., Zhou H., Qu L.H., Yang J.H. (2014). StarBase v2.0: Decoding MiRNA-CeRNA, MiRNA-NcRNA and Protein–RNA Interaction Networks from Large-Scale CLIP-Seq Data. Nucleic Acids Res..

[42] Wa Q., Li L., Lin H., Peng X., Ren D., Huang Y., He P., Huang S. (2018). Downregulation of MiR-19a-3p Promotes Invasion, Migration and Bone Metastasis via Activating TGF-β Signaling in Prostate Cancer. Oncol. Rep..

[43] Lee S., Lee H., Bae H., Choi E.H., Kim S.J. (2016). Epigenetic Silencing of MiR-19a-3p by Cold Atmospheric Plasma Contributes to Proliferation Inhibition of the MCF-7 Breast Cancer Cell. Sci. Rep..

[44] Mearini E., Poli G., Cochetti G., Boni A., Egidi M.G., Brancorsini S. (2017). Expression of urinary miRNAs targeting NLRs inflammasomes in bladder cancer. Onco. Targets Ther..

[45] Wang F., Li T., Zhang B., Li H., Wu Q., Yang L., Nie Y., Wu K., Shi Y., Fan D. (2013). MicroRNA-19a/b Regulates Multidrug Resistance in Human Gastric Cancer Cells by Targeting PTEN. Biochem. Biophys. Res. Commun..

[46] Han Y., Chen J., Zhao X., Liang C., Wang Y., Sun L., Jiang Z., Zhang Z., Yang R., Chen J. (2011). MicroRNA Expression Signatures of Bladder Cancer Revealed by Deep Sequencing. PLoS ONE.

[47] Ratert N., Meyer H.-A., Jung M., Lioudmer P., Mollenkopf H.-J., Wagner I., Miller K., Kilic E., Erbersdobler A., Weikert S. (2013). MiRNA Profiling Identifies Candidate MiRNAs for Bladder Cancer Diagnosis and Clinical Outcome. J. Mol. Diagn..

[48] Gu Y., Liu S., Zhang X., Chen G., Liang H., Yu M., Liao Z., Zhou Y., Zhang C.-Y., Wang T. (2017). Oncogenic MiR-19a and MiR-19b Co-Regulate Tumor Suppressor MTUS1 to Promote Cell Proliferation and Migration in Lung Cancer. Protein Cell.

[49] Xu X.-L., Jiang Y.-H., Feng J.-G., Su D., Chen P.-C., Mao W.-M. (2014). MicroRNA-17, MicroRNA-18a, and MicroRNA-19a Are Prognostic Indicators in Esophageal Squamous Cell Carcinoma. Ann. Thorac. Surg..

[50] Torres-Bustamante M.I., Vazquez-Urrutia J.R., Solorzano-Ibarra F., Ortiz-Lazareno P.C. (2024). The Role of MiRNAs to Detect Progression, Stratify, and Predict Relevant Clinical Outcomes in Bladder Cancer. Int. J. Mol. Sci..

