# Supplementary material for: Association of miRNA-17-92 Cluster with Muscle Invasion in Bladder Cancer
Source: Int J Mol Sci. 2025 Aug 5;26(15):7546. doi: 10.3390/ijms26157546 (PMC12347747; doi:10.3390/ijms26157546)
Supplement: Supplementary file 1 [file ijms-26-07546-s001.zip › ijms-3762340-supplementary.pdf]

Supplementary Table 2. miRNAs differentially expressed in our case-control study (Mann-Whitney test) and confirmed in public dataset (GSE40355).

miRNA	Case-control (41 T *vs* 28 PT) (our study)		Case-control (16 T *vs* 8 CTRL) (GSE40355)	
	**FR**	**p-value**	**FR**	**p-value**
**miR-133a-3p**	**-13.49**	<0.001	**-769.02**	<0.001
**miR-1**	**-9.20**	<0.001	**-642.75**	<0.001
**miR-100-5p**	**-8.12**	<0.001	**-80.00**	0.001
**miR-99a-5p**	**-7.95**	<0.001	**-166.50**	<0.001
**miR-125b-5p**	**-7.15**	<0.001	**-75.39**	<0.001
**miR-145-5p**	**-6.84**	<0.001	**-46.55**	<0.001
**miR-143-3p**	**-5.47**	<0.001	**-717.83**	<0.001
**miR-132-3p**	**-3.37**	<0.001	**-95.42**	<0.001
**miR-150-5p**	**-3.20**	<0.001	**-6.08**	ns
**miR-214-3p**	**-2.86**	<0.001	**-48.06**	<0.001
**miR-126-3p**	**-2.35**	<0.001	**-1.5**	ns
**let-7c-5p**	**-2.35**	<0.001	**1.87**	0.001
**miR-195-5p**	**-2.17**	<0.001	**-6.69**	<0.001
**miR-26a-5p**	**-2.02**	<0.001	**-2.49**	<0.001
**let-7i-5p**	**-2.00**	<0.001	**1.87**	0.001
**miR-200c-3p**	**2.05**	0.018	**16.32**	<0.001
**miR-106b-5p**	**2.13**	0.003	**3.84**	<0.001
**miR-20a-5p**	**2.23**	0.011	**2.78**	<0.001
**miR-106a-5p**	**2.33**	0.039	**1.87**	0.001
**miR-21-5p**	**2.51**	0.004	**1.94**	0.049
**miR-19b-3p**	**2.75**	0.006	**3.39**	0.001
**miR-17-5p**	**3.00**	0.001	**2.79**	0.001
**miR-19a-3p**	**3.09**	0.003	**5.10**	0.001
**miR-18a-5p**	**3.55**	<0.001	**1.87**	0.001
**miR-182-5p**	**4.79**	<0.001	**121.89**	<0.001
**miR-210-3p**	**5.38**	<0.001	**8.96**	<0.001
